# A Coarse-Grained Molecular Dynamics Model for Analysis of Mesoscale Carbon Nanothread Structures

**DOI:** 10.3390/polym18172114

**Published:** 2026-08-31

**Authors:** Jiajia Cheng, Junshan Si, Nan Wu, Jun Liu, Su Ju, Yonglyu He, Jianwei Zhang, Ke Duan

**Affiliations:** College of Aerospace Science and Engineering, National University of Defense Technology, Changsha 410073, China

**Keywords:** diamond nanothreads, coarse-grained model, mechanical behavior, molecular dynamics simulations

## Abstract

Diamond nanothreads (DNTs) represent a promising class of one-dimensional carbon nanomaterials for next-generation structural applications. However, exploring their mesoscale collective properties remains computationally prohibitive via all-atomistic molecular dynamics (MD) simulations. Here, we present a physically consistent coarse-grained model tailored for both zigzag (DNT-I) and tubular (DNT-II) nanothreads. By establishing an energy equivalence framework between all-atomistic MD simulations and molecular mechanics, the bonded potentials (stretching and bending) and non-bonded Lennard-Jones parameters were derived. Moreover, a degree of coarse-graining *r*_0_ = 6 Å was determined, which well preserves the interfacial cohesive energy and axial sliding behavior of all-atomistic models. Using the established coarse-grained potentials of DNTs, a high glass transition temperature (*T*_g_ = 1485 K) was predicted, and a cooperative intermolecular sliding mechanism that governs the plastic deformation of crystalline DNT aggregates under uniaxial tension was revealed.

## 1. Introduction

Diamond nanothreads (DNTs), fully *sp*^3^-bonded one-dimensional carbon allotropes first synthesized via the high-pressure solid-state reaction of crystalline benzene [1], have emerged as a new class of nanomaterials for advanced structural and functional applications. Different from *sp*^2^-hybridized carbon nanotubes (CNTs), DNTs possess a fully hydrogenated structural morphology [2,3] and exhibit an intermediate bending rigidity that bridges highly flexible organic polymer chains and ultra-rigid CNTs due to their ultra-small diameters (4–6 Å) [4,5]. This intermediate bending rigidity allows DNT aggregates to self-assemble into extremely dense networks without undergoing the catastrophic radial collapse or structural flattening commonly observed in CNT bundles under deformation [6]. Consequently, DNTs hold exceptional potential for reinforcing temperature-resistant, lightweight macroscopic materials that far surpass the performance limits of conventional polymers.

Due to their outstanding mechanical properties and non-smooth hydrogenated surfaces, DNTs show immense promise as reinforcing fillers in high-performance polymer nanocomposites [7,8,9]. Interfacial pull-out evaluations demonstrate that DNT bundles exhibit exceptional interfacial load-transfer efficiency, yielding an interfacial shear strength that is nearly an order of magnitude higher than that of pristine CNTs due to strong mechanical interlocking of their corrugated surfaces [6]. However, the macroscopic reinforcing efficiency of DNTs is highly sensitive to filler dispersion and localized interfacial load-transfer characteristics. On one hand, randomly dispersed DNTs tend to introduce continuous lower-density interphase zones that can degrade the composite modulus [10]. Although this degradation can be mitigated by surface functionalization or interfacial cross-linking [10], the actual reinforcement is highly anisotropic and relies heavily on the loading scenarios. Specifically, the mechanical enhancement is highly pronounced under simultaneous matrix-filler co-deformation but is significantly suppressed under non-simultaneous or lateral deformation, where the load transfer is dominated by local non-covalent interactions [11].

Serving as the fundamental building blocks for macro-aggregates and nanocomposites, a comprehensive understanding of their collective physical properties, such as the glass transition temperature (*T*_g_) and large-deformation behaviors, is essential. However, experimentally characterizing these collective properties remains a formidable challenge, primarily due to the microgram-scale synthetic yields. Consequently, atomic-level molecular dynamics (MD) simulations have become an indispensable approach to predict and understand their thermal and mechanical behaviors. Nevertheless, conventional all-atomistic (AA) MD simulations are computationally prohibitive for large-scale systems containing thousands of polymer chains and DNT monomers. This massive computational cost hinders the direct investigation of mesoscale phenomena, such as aggregate self-assembly, thermodynamic transitions, and progressive failure propagation.

To bridge the gap between atomistic interactions and macroscopic performance, developing accurate and robust coarse-grained (CG) models has become an attractive approach [12,13,14,15,16,17,18,19]. Among traditional bottom-up methods, Iterative Boltzmann Inversion (IBI) is a structure-based approach that optimizes potentials by matching all-atomistic pair distribution functions, making it suitable for flexible polymers. In contrast, Force Matching (FM) and energy-equivalence schemes fit CG forces or energy landscapes directly to AA trajectories, providing an accurate description of rigid structural backbones. However, when applying force/energy matching schemes to one-dimensional (1D) carbon materials (such as CNTs), existing CG models often treat the interface simplistically using standard Lennard-Jones (LJ) potentials with parameters derived from arithmetic means. Furthermore, the degree of coarse-graining in these models is typically chosen arbitrarily without rigorous evaluation of its physical validity. In DNT systems, this simplification is particularly problematic; DNTs possess discrete, highly corrugated hydrogenated surfaces that dictate unique non-bonded energy landscapes. Neglecting the characteristic repeating unit length and the discrete lattice registry will inevitably cause the CG model to either overestimate surface roughness (leading to artificial interlocking) or artificially smooth out the axial sliding energy barriers. Consequently, developing a physically consistent CG model that concurrently preserves the intramolecular mechanics and the intermolecular registry-matching remains a critical challenge.

In this work, we developed a physically consistent CG potential for both zigzag (DNT-I) and tubular (DNT-II) diamond nanothreads. Combining AA MD simulations with molecular mechanics, the bonded potentials (stretching and bending) were first derived from continuum beam and rod models. The non-bonded potentials were then established by reproducing both the thermodynamic cohesive energy and the shear-sliding characteristic of the AA models, yielding a degree of coarse-graining of *r*_0_ = 6 Å. Finally, the newly developed CG potentials were applied to construct a mesoscale aggregate model, predicting a high glass transition temperature of 1485 K, which substantially surpasses the thermal limits of conventional polymer materials. Moreover, a cooperative intermolecular sliding mechanism that governs the plastic deformation of crystalline DNT aggregates under uniaxial tension was revealed. This study provides an efficient and reliable modeling tool to facilitate the design of DNT-based advanced materials.

## 2. Methods

### 2.1. Atomistic Configurations of DNT-I and DNT-II

**Figure 1:**
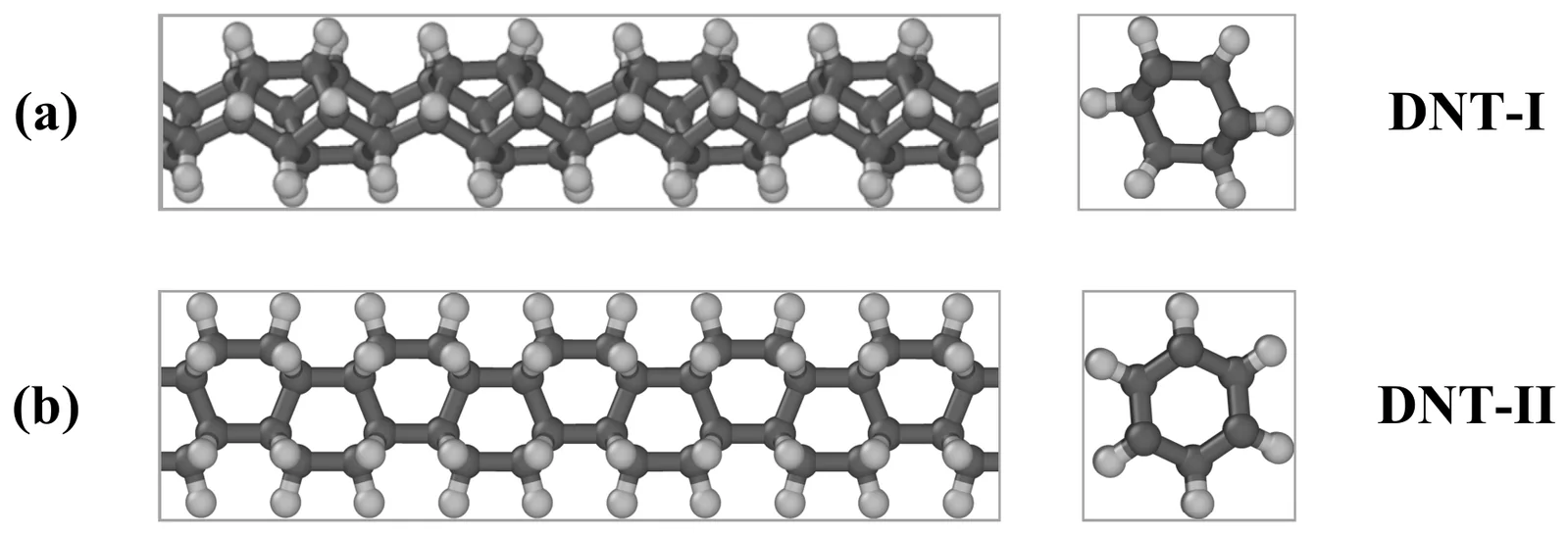
All-atomistic configurations of the DNTs: (**a**) DNT-I and (**b**) DNT-II. The left shows a view perpendicular to the axis, and the right shows a cross-sectional view. Dark gray and light gray spheres represent carbon (C) and hydrogen (H) atoms, respectively.

Diamond nanothreads are constructed by systematically varying the topology of *sp*^3^ bond pathways between precursor benzene rings [3]. In this work, two distinct straight DNTs, the zigzag-shaped DNT-I and the tubular-shaped DNT-II, are investigated as representatives [2,20]. These two structures represent the major DNT topologies that have been successfully synthesized and structurally characterized in experimental studies [1]. Moreover, their straight and geometrically symmetric morphologies provide an ideal benchmark for deriving and calibrating the 1D coarse-grained potentials before applying them to more complex helical configurations. The optimized molecular structures of these two DNTs are shown in Figure 1. To ensure a rigorous basis, both DNT models are constructed with an identical number of carbon atoms. Due to the presence of Stone–Wales (SW) defects in the zigzag skeleton, the equilibrium length of the DNT-I model is 75.29 Å, whereas the DNT-II model has a length of 64.93 Å. The adaptive intermolecular reactive empirical bond order (AIREBO) potential [21,22] is employed to describe the intramolecular covalent (C–C, C–H) and intermolecular non-bonded dispersive interactions. The covalent interaction cutoff radius is set to 2.0 Å. To suppress the influence of thermal fluctuations on the static elastic and adhesive property calibrations, all characterization simulations are conducted at a cryogenic temperature of 1 K [4,23]. AA-MD simulations are performed using the Large-scale Atomic/Molecular Massively Parallel Simulator (LAMMPS) package [24].

### 2.2. AA MD Simulations of DNT Models

To construct and parameterize a physically consistent coarse-grained (CG) force field, three types of AA-MD simulations were performed: (1) uniaxial tensile simulations to characterize the axial stiffness, (2) pure bending simulations to determine the out-of-plane bending rigidity, and (3) radial adhesion tests to map the intermolecular van der Waals (vdW) cohesive energy landscape.

#### 2.2.1. Uniaxial Tensile Simulations

**Figure 2:**
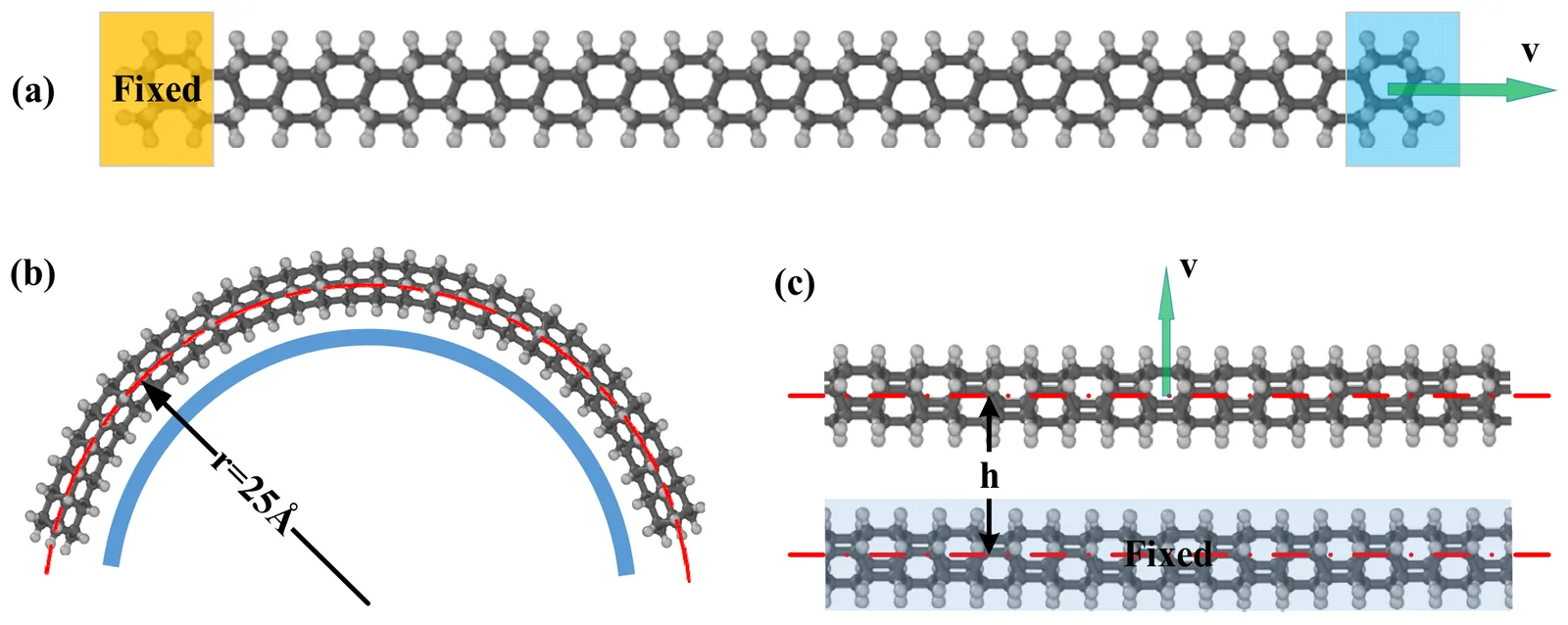
Schematic view of simulation protocols for DNT-II. (**a**) Tensile simulation of DNT, (**b**) bending simulation scheme of DNT with a curvature of 0.025 Å^−1^ introduced by an imaginary sphere LJ wall, and (**c**) adhesion test achieved by keeping the lower DNT fixed and moving the upper DNT.

To evaluate the axial stretching characteristics, the constructed DNT models are first subjected to energy minimization using the conjugate gradient algorithm [9,25]. Subsequently, the structures are fully equilibrated in the canonical ensemble (NVT) at 1 K using a Nosé–Hoover thermostat for 10 ns with an integration timestep of 1 fs [26,27]. Uniaxial tensile loading is then implemented by fixing the atoms at one end of the nanothread along the axial direction while displacing the opposite end quasi-statically at a constant displacement rate of 0.005 Å/ps, as shown in Figure 2a. The dynamic deformation trajectory is tracked, and the simulation is terminated upon the onset of structural rupture. The instantaneous axial stress is calculated via the virial stress tensor based on the effective volume of the DNT tube [4], where the outer radius of the cross-section is assumed to be 2.5 Å [5]. The axial strain is directly derived from the displacement of the loading boundary. Finally, the Young’s modulus (*E*) is determined by fitting the linear elastic regime of the resulting stress–strain curve within a small-strain limit (≤3%).

#### 2.2.2. Pure Bending Simulations

Owing to their ultra-small diameters and intermediate rigidity, DNTs exhibit distinct bending compliance and cannot be treated as simplified rigid rods. To achieve pure bending deformation without introducing localized buckling or shear stress [13], we implement a virtual spherical Lennard-Jones (LJ) 9/3 wall with a systematically varied curvature ($k=1/R_{\mathrm{wall}}$), as illustrated in Figure 2b. The non-bonded interaction between the virtual wall and the DNT atoms is governed by a conformal LJ-9/3 potential [4]:

(1) $$U_{\mathrm{wall}}=\varepsilon_{\mathrm{wall}}\left[\frac{2}{15}{\left(\frac{\sigma_{\mathrm{wall}}}{r}\right)}^{9}-{\left(\frac{\sigma_{\mathrm{wall}}}{r}\right)}^{3}\right]$$

where $\varepsilon_{\mathrm{wall}}$ represents the interaction strength, $\sigma_{\mathrm{wall}}$ is the van der Waals distance parameter, and *r* denotes the shortest perpendicular distance from a DNT atom to the virtual wall boundary.

Following an initial 10 ns relaxation in the NVT ensemble, the virtual “wall-atom” LJ interaction is activated, forcing the flexible DNT to gradually combine and conform to the spherical wall surface. The system is relaxed for an additional 5 ns under NVT conditions to ensure structural and thermodynamic stability. Once completely relaxed, the bending strain energy (*E*_b_) is calculated as the potential energy difference between the bent DNT and the unbent ground state. According to classical continuum beam theory, the elastic bending energy per unit length is formulated as [28]:

(2) $$\frac{2E_{b}}{L}=(EI)k^{2}$$

where *EI* is the bending stiffness, *L* is the equilibrium length of the DNT, and *k* is the prescribed curvature [28]. By fitting the normalized strain energy $\frac{2E_{b}}{L}$ against *k*^2^ across a range of curvature, the bending stiffness *EI* can be determined.

#### 2.2.3. Radial Adhesion Test

**Figure 3:**
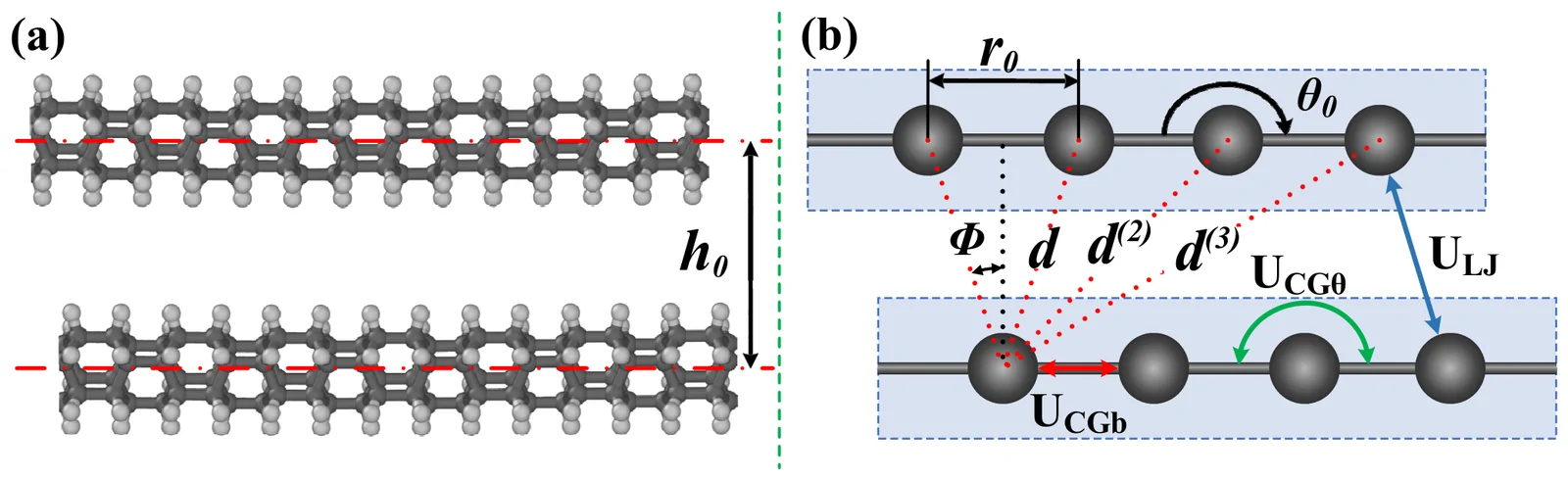
The all-atomistic structure and coarse-grained model for two parallel DNT-II. (**a**) All-atomistic model; (**b**) the coarse-grained model including bonded and non-bonded interacting details.

The intermolecular adhesion properties dictate the packing behavior and physical interactions of multiple parallel DNTs. To investigate the cohesive energy landscape, we establish configurations consisting of two parallel, identical DNTs, as shown in Figure 2c. The simulations are conducted in the isothermal-isobaric (NPT) ensemble at 1 K with periodic boundary conditions applied along the axial direction to eliminate end-edge effects. During the adhesion tests, as shown in Figure 3a, the atoms of the lower DNT are held fixed, while the upper DNT is displaced quasi-statically to systematically vary the center-to-center radial separation distance (*h*). By recording the total potential energy of the system as a function of *h*, the non-bonded van der Waals interaction energy landscape is mapped, yielding a potential energy per unit length ($U_{L}$) curve that directly calibrates the non-bonded cohesive parameters for the downstream coarse-grained model [29,30,31], as shown in Figure 3b.

## 3. Results and Discussion

### 3.1. Analytical Derivation of Coarse-Grained Bonded Potentials

The parameterization of the DNT CG potentials is based on the principle of energy equivalence between the discrete bead-spring representation and classical continuum mechanics [12,29,30]. For a one-dimensional nanothread, the total bonded strain energy $U_{\mathrm{bonded}}$ is decoupled into axial stretching $U_{\mathrm{stretch}}(r)$ and out-of-plane bending $U_{\mathrm{bend}}(\theta )$ components:

(3) $$U_{\mathrm{bonded}}=U_{\mathrm{stretch}}(r)+U_{\mathrm{bend}}(\theta )$$

(4) $$U_{\mathrm{stretch}}(r)=\sum_{i=1}^{m}k_{b}(r_{i}-r_{0}{)}^{2}$$

(5) $$U_{\mathrm{bend}}(\theta )=\sum_{j=1}^{n}k_{\theta }(\theta_{j}-\theta_{0}{)}^{2}$$

where *r_i_* and *θ_j_* represent the instantaneous bond length and bending angle of adjacent CG beads (shown in Figure 3b), *r*_0_ is the degree of coarse-graining, which is equal to the distance between two beads [32], and *θ*_0_ is the equilibrium bending angle (180°). The parameters *k_b_* and *k_θ_* represent the harmonic stretching and bending stiffness coefficients.

According to classical continuum beam and rod theories, the strain energy of a uniform elastic cylinder of length *L* subjected to pure tension (∆*L*) and pure bending (end rotation angle Δϕ) is analytically expressed as [33]:

(6) $$U_{\mathrm{stretch}}^{\mathrm{cont}}=\frac{EA}{2L}{\left(\Delta L\right)}^{2}$$

(7) $$U_{\mathrm{bend}}^{\mathrm{cont}}=\frac{EI}{2L}{\left(\Delta \phi \right)}^{2}$$

where *EA* represents the axial tensile stiffness and *EI* represents the bending stiffness. As a result, the harmonic stiffness coefficients can be calculated by:

(8) $$k_{b}=\frac{EA}{r_{0}}$$

(9) $$k_{\theta }=\frac{EI}{r_{0}}$$

**Figure 4:**
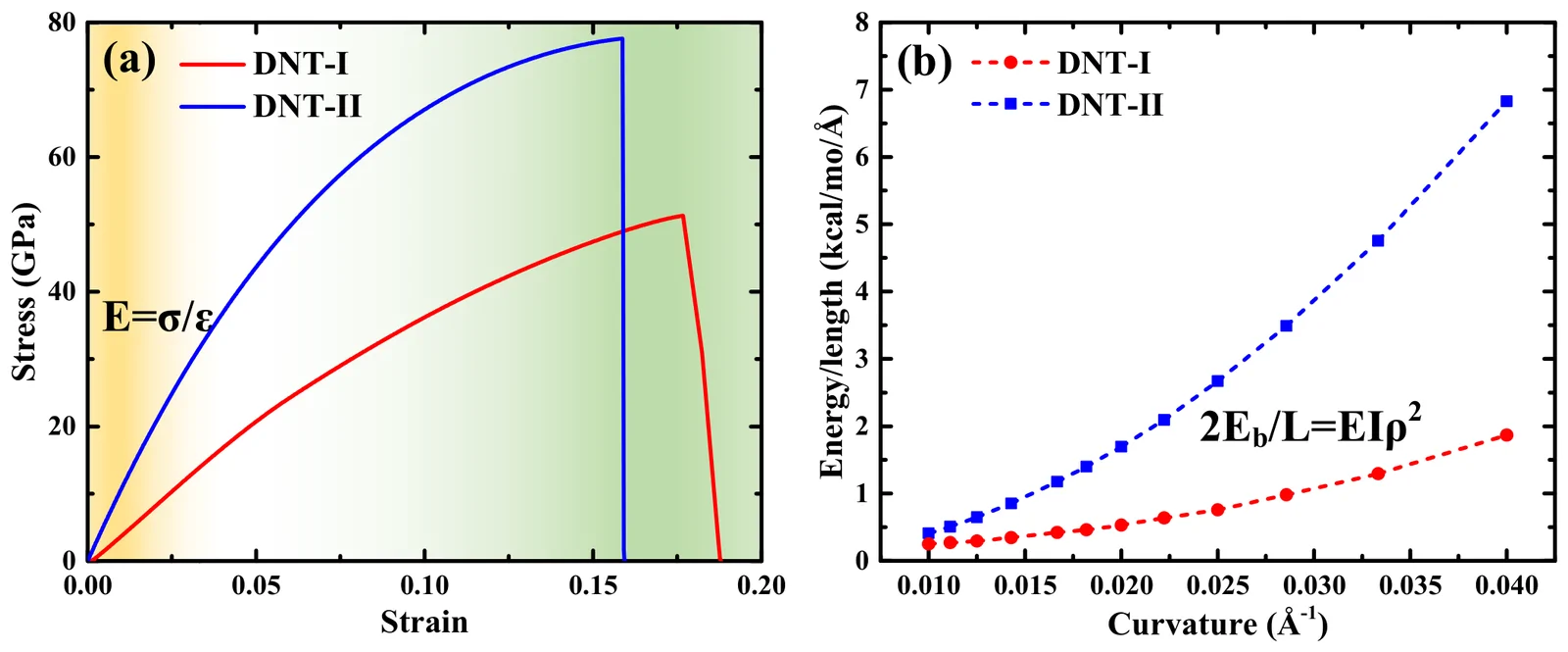
Comparison of the bond mechanical properties of all-atomistic DNT-I and DNT-II: (**a**) a stress–strain curve and (**b**) bending strain energy *E_b_* normalized by sample length 2*E_b_*/*L* as a function of the curvature.

To calibrate the macroscopic elastic parameters (*EA* and *EI*), AA-MD simulations were performed. Figure 4a displays the uniaxial stress–strain responses of DNT-I and DNT-II. Within the small-strain elastic regime (strain is less than 3%), the fitted Young’s modulus is 404.5 GPa for the zigzag DNT-I and 1104.4 GPa for the tubular DNT-II. Under pure bending states, as shown in Figure 4b, the normalized bending strain energy ($\frac{2E_{b}}{L}$) exhibits a quadratic relationship with the prescribed curvature. The calculated bending stiffness *EI* is 1289.6 kcal/mol·Å for DNT-I and 4241.0 kcal/mol·Å for DNT-II, reflecting the higher intrinsic rigidity of the tubular *sp*^3^ network compared to its zigzag counterpart, as listed in Table 1.

### 3.2. Formulations of Coarse-Grained Non-Bonded Potentials

Intermolecular non-bonded interactions govern the packing structure and load-transfer capacity of DNT aggregates. In our CG framework, the pairwise non-bonded dispersive interactions are described using a classical Lennard-Jones (12-6) potential:

(10) $$U_{LJ}\left(d\right)=4\varepsilon \left[{\left(\frac{\sigma }{d}\right)}^{12}-{\left(\frac{\sigma }{d}\right)}^{6}\right]$$

where *d* is the distance between the interacting beads, *ε* is the potential energy well depth, and *σ* represents the zero-energy distance.

To establish the non-bonded parameters analytically, we develop a 1D staggered parallel-chain model (Figure 3b). Under thermodynamic equilibrium, the radial separation distance between two parallel DNTs is *h*_0_. The equilibrium distance (*d*) between the nearest interacting CG beads and the corresponding staggered angle (ϕ) are geometrically formulated as:

(11) $$d=\frac{h_{0}}{\cos\phi }$$

Angle *ϕ* can be derived from the geometry of the two parallel CG DNTs,

(12) $$\phi =\arctan\left(\frac{r_{0}}{2h_{0}}\right)$$

where *r*_0_ represents the equilibrium bond length of the CG beads, as shown in Figure 3b.

The total vdW cohesive energy per unit length ($U_{L}$) of the two parallel DNTs is calculated by summing the pairwise interactions along the staggered chains. Following Buehler’s formulation [13], the long-range interactions are truncated at the sixth neighbor beads. By evaluating the energy minimization condition of the nearest-neighbor pair ($U_{LJ}(d)=-\varepsilon$), the total cohesive energy is simplified as:

(13) $$U_{L}(h_{0})=-\frac{2\beta \varepsilon }{r_{0}}$$

where β is a dimensionless scaling factor representing the contribution of higher-order neighbor beads and equals 1.0988 [13].

By decoupling Equation (13) and the zero-force condition, the CG non-bonded parameters *ε* and *σ* are analytically solved as:

(14) $$\varepsilon =-\frac{U_{L}r_{0}}{2.1976}$$

(15) $$\sigma =\frac{h_{0}}{\sqrt[{6}]{2}\cos\phi }$$

**Figure 5:**
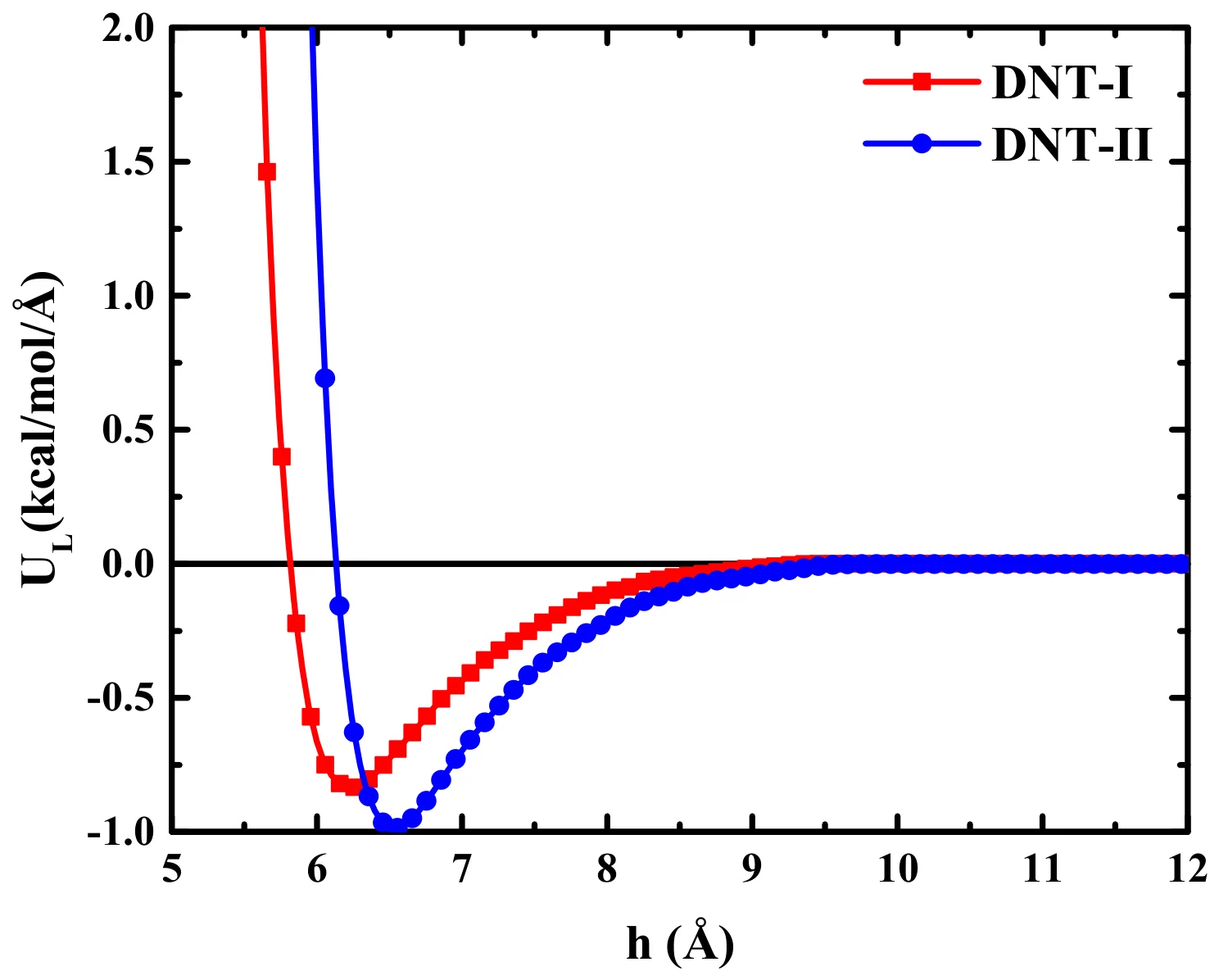
Comparison of the cohesive energy per unit length versus different distances between two parallel DNTs using an all-atomistic model of DNT-I and DNT-II.

Through AA-MD radial adhesion tests, as shown in Figure 5, the radial equilibrium distance *h*_0_ is determined to be 6.25 Å and 6.55 Å for parallel DNT-I and DNT-II pairs, yielding cohesive energies ($U_{L}$) of −0.8331 kcal/mol/Å and −0.9874 kcal/mol/Å, respectively. Based on these AA benchmarks, the analytical CG potential parameters are determined as functions of the chosen degree of coarse-graining *r*_0_ and are listed in Table 2 and Table 3.

### 3.3. Determination of the Degree of Coarse-Graining r_0_

**Figure 6:**
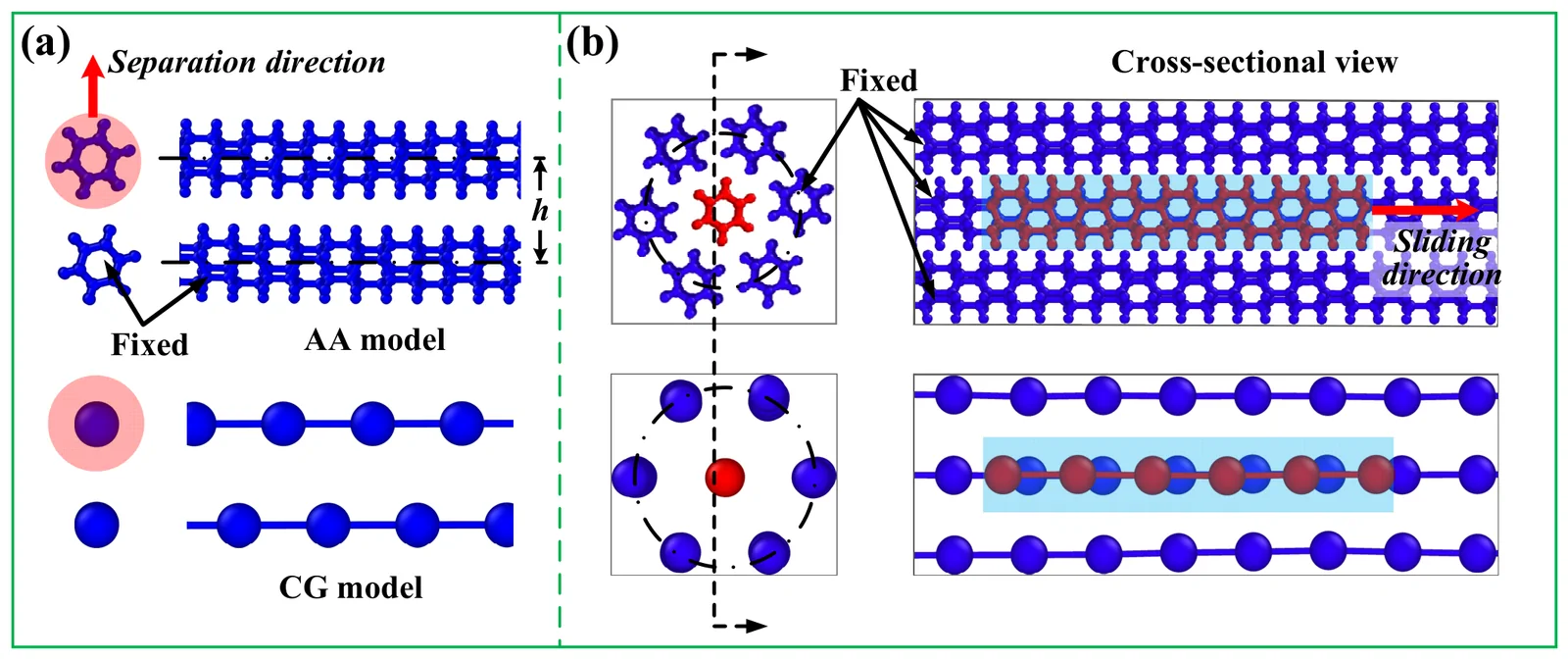
(**a**) Separation test of two parallel DNTs; (**b**) sliding test of DNT bundle.

The selection of a proper degree of coarse-graining (*r*_0_) is a key step in developing consistent CG potentials. The CG potentials should be able to capture the interfacial interactions between neighboring DNTs (Figure 6) to accurately simulate the mechanical behavior of DNT aggregates. Typically, these interactions are determined by the normal separation and axial shear between the threads [31,32]. Because the choice of *r*_0_ directly changes the spatial resolution and the non-bonded potentials of the CG beads, we varied the value of *r*_0_ from 2 Å to 8 Å to find the optimal value.

**Figure 7:**
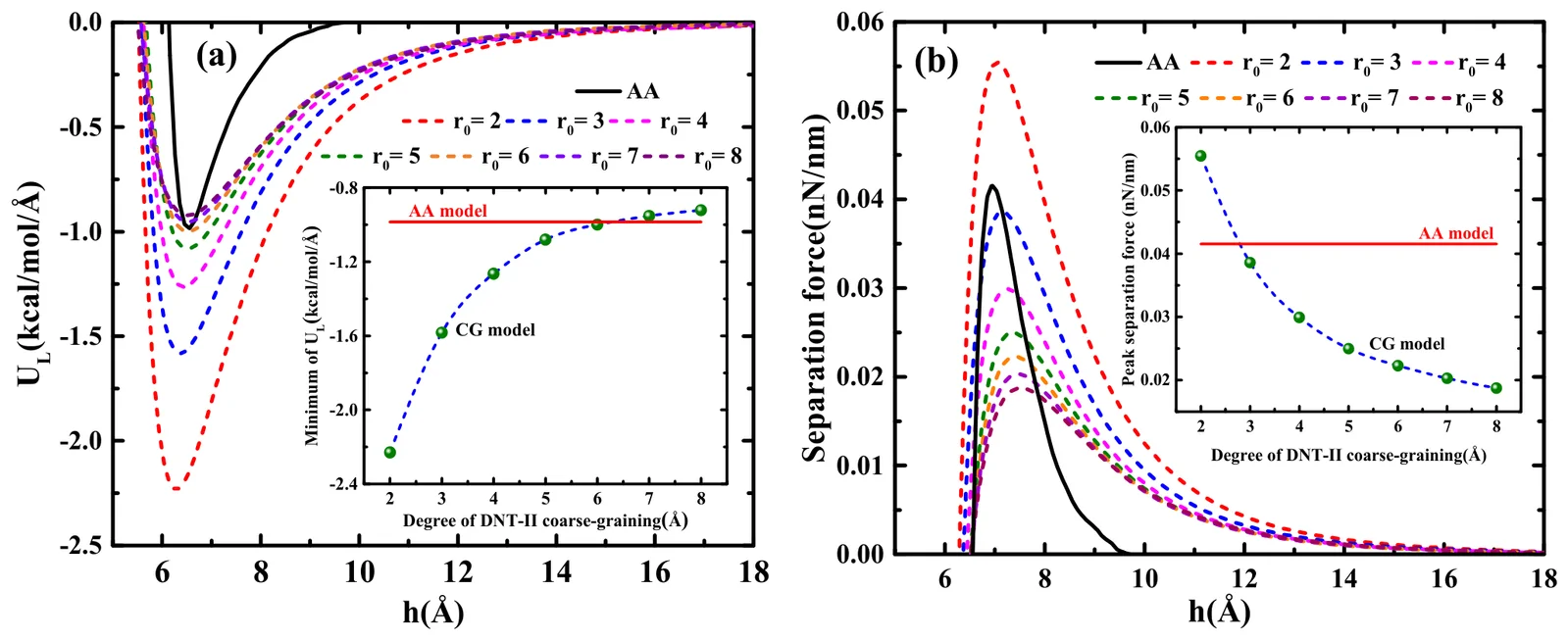
(**a**) The vdW energy per unit length *U_L_* and (**b**) separation force versus different distances between two parallel DNT-II of AA and CG models of the separation test.

**Figure 8:**
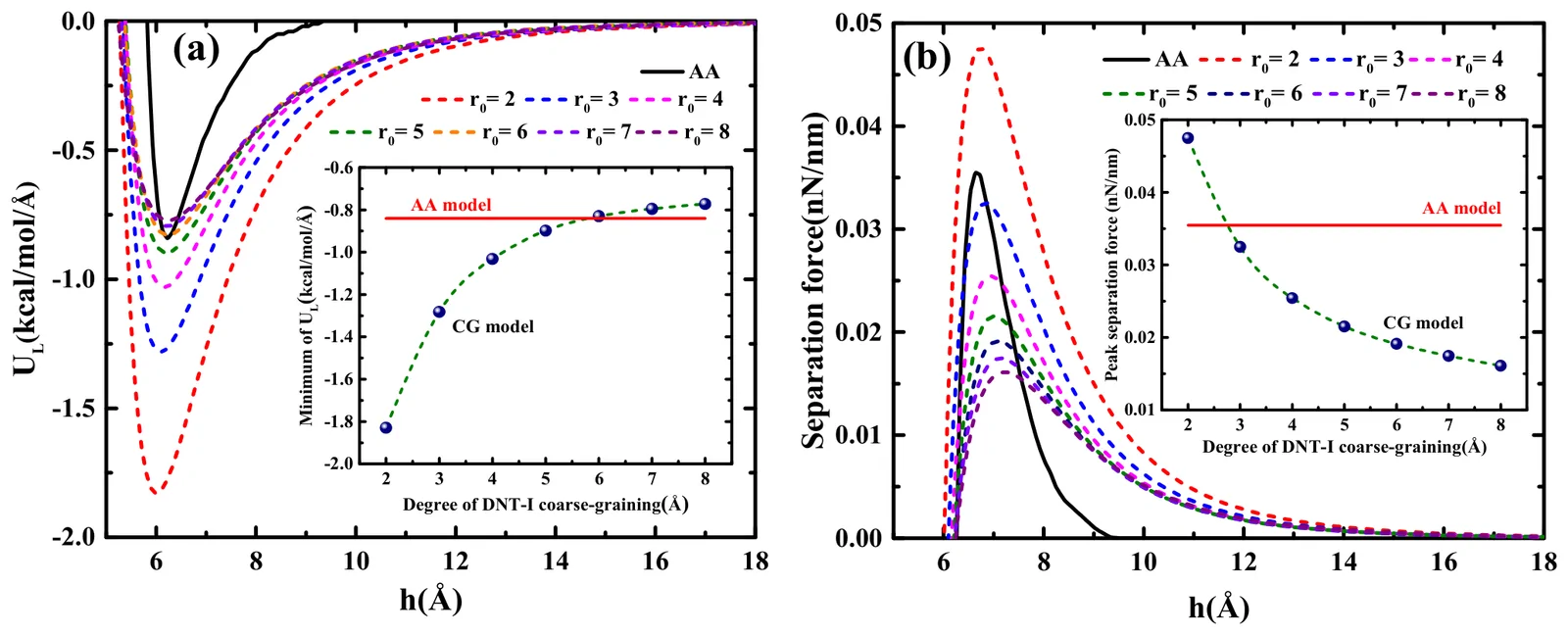
(**a**) The vdW energy per unit length *U_L_* and (**b**) separation force versus different distances between two parallel DNT-I of AA and CG models of the separation test.

The variation of cohesive energy and separation force of DNTs with different degrees of coarse-graining in normal separation for DNT-II and DNT-I is shown in Figure 7 and Figure 8, respectively. It can be observed that the cohesive energy per unit length (*U_L_*) of all CG models aligns at the same radial equilibrium distance *h*_0_ (6.25 Å for DNT-I and 6.55 Å for DNT-II). However, the minimum value of *U_L_* is sensitive to *r*_0_. For DNT-II, the cohesive energy of the CG model at *r*_0_ = 6 Å agrees well with its AA counterpart (−0.985 kcal/mol/Å). In contrast, for the condition of *r*_0_ = 6 Å, the peak separation force of CG models generally underestimated the peak force of AA models. The best match for the peak separation force occurs at a smaller *r*_0_ = 3 Å. This reveals a clear mismatch, namely the optimal *r*_0_ derived from the cohesive energy is different from the one derived from the separation force. This force-energy discrepancy is a common limitation in 1D coarse-graining, where grouping multiple atoms into a single bead smooths out the local force gradients [32].

**Figure 9:**
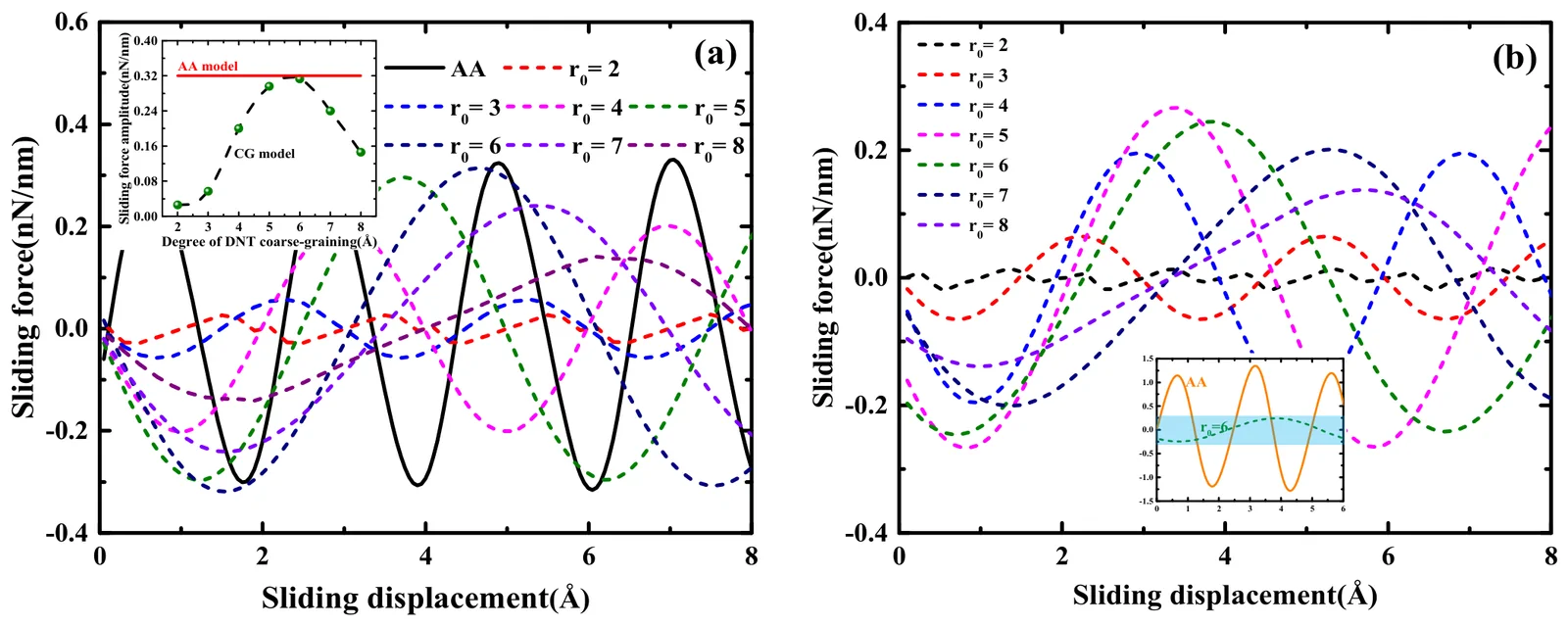
Curve of the sliding force as a function of sliding displacement of (**a**) DNT-II and (**b**) DNT-I.

The interfacial sliding force as a function of sliding displacement for different degrees of coarse-graining is displayed in Figure 9. Due to the periodic structure of the DNT lattice, the sliding forces of both AA and CG models show regular sine-wave oscillations. As shown in the inset of Figure 9a, the sliding force amplitude of DNT-II changes non-linearly with *r*_0_ and reaches a peak of 0.313 nN/nm at *r*_0_ = 6 Å, which is in excellent agreement with the AA sliding force amplitude (0.32 nN/nm). For DNT-I, as shown in Figure 9b, the amplitude of the sliding force of the AA model is about 1.3 nN/nm, which is significantly higher than the maximum value of all CG models, demonstrating that the real interfacial behavior is difficult to fully reproduce by CG potentials for DNT-I.

To resolve the conflict between these optimal parameters and determine the most reasonable degree of coarse-graining, we select *r*_0_ = 6 Å as the standard parameter for both DNT-I and DNT-II based on three physical and practical considerations. First, the cohesive energy of DNTs is the key factor that controls the stable packing structure, density, and thermal properties (such as the glass transition temperature, *T*_g_) of DNT aggregates [31]. Choosing *r*_0_ = 6 Å preserves this critical cohesive energy, ensuring that the simulated aggregate is thermodynamically stable and structurally correct. Second, when DNT aggregates are subjected to external loads, the main deformation and energy dissipation mechanisms are dominated by the relative axial sliding (shear) between the parallel threads, rather than radial separation [6,11]. In a closely packed aggregate, the radial separation of DNTs is strongly restricted by surrounding neighbor threads, making the normal separation force much less critical than the axial sliding force during deformation. Because *r*_0_ = 6 Å offers the best match for the sliding force amplitude of DNT-II and provides the maximum sliding barrier for DNT-I among all CG candidates, it is the most suitable parameter to predict the tensile and shear properties of DNT aggregates. Third, selecting a uniform *r*_0_ = 6 Å for both nanothreads maintains model consistency for future multi-component simulations while providing a massive computational speedup compared to the AA models. Therefore, *r*_0_ = 6 Å is determined as the optimal degree of coarse-graining to investigate the thermomechanical properties of DNT aggregates, and the derived CG potential parameters are summarized in Table 4. It should be noted that the current potentials in Table 4 are derived from pristine DNTs, and a direct recalibration via AA-MD is required if high-density defects are present.

## 4. Discussion

**Figure 10:**
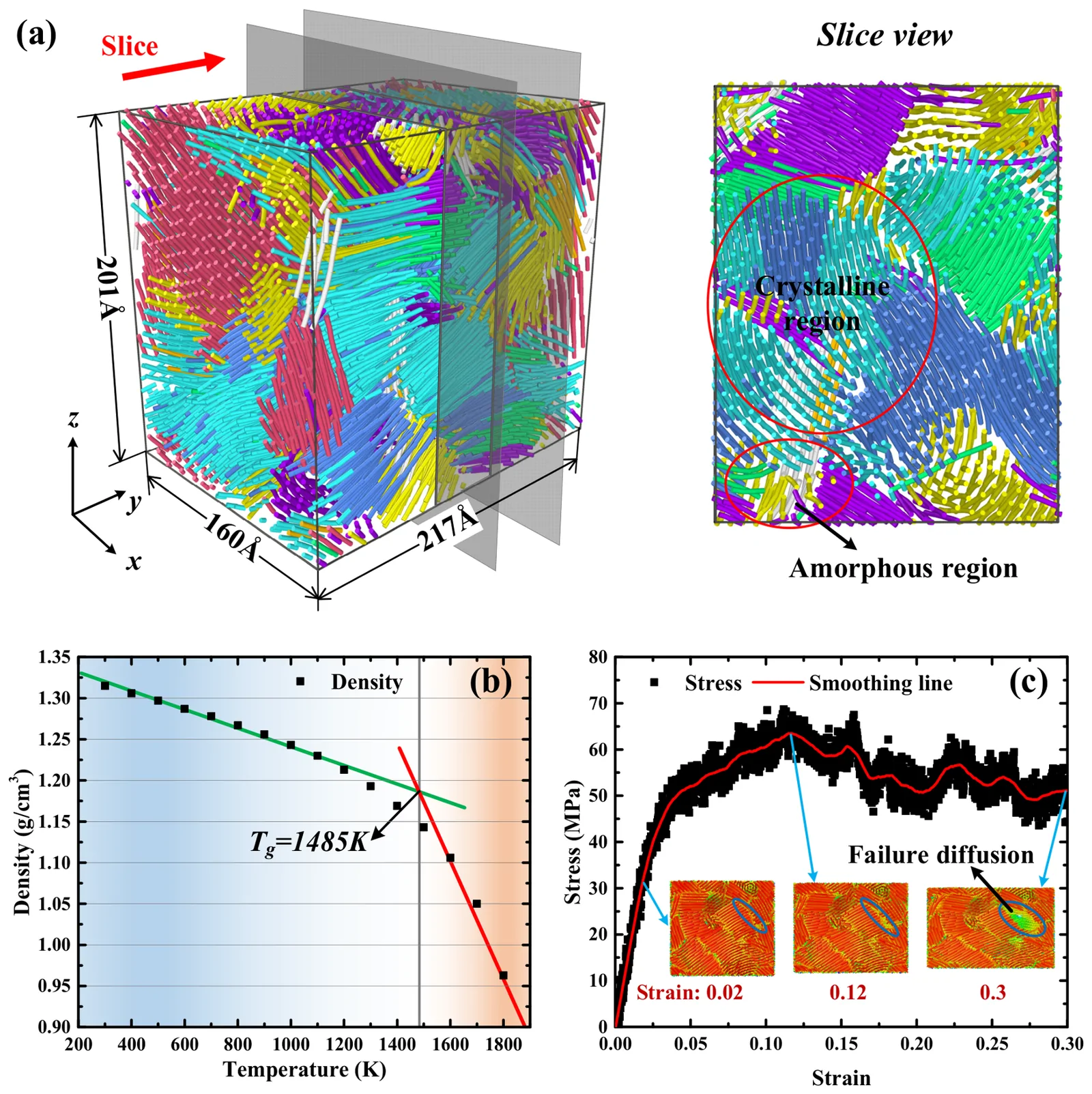
(**a**) Representative volume elements (RVE) for the DNT-I aggregate after the annealing equilibrium process; (**b**) the density of the annealing process is plotted as a function of temperature; (**c**) the strain–stress curves under uniaxial tension.

In this section, we construct a mesoscale model to investigate the packing structure and mechanical properties of DNT-I aggregates based on the developed CG potentials (the potential functions and parameters are compiled in Table 4). An initial amorphous structure of randomly distributed DNTs is generated at a low density of 0.2 g/cm^3^. The system contains a total of 30,000 beads, representing 3000 individual DNT-I chains with 10 beads per chain (equivalent to a length of 6 nm per chain). This initial structure is further annealed from 1800 K to 300 K under the NPT ensemble (pressure 1 atm) until thermodynamic equilibrium is reached. Specifically, the annealing process consists of 14 cooling cycles over a total duration of 7 ns. In each cycle, the system is cooled by 100 K in 50 ps and then kept at that temperature for 450 ps. The equilibrated system exhibits a crystalline packing with a density of 1.316 g/cm^3^ as shown in Figure 10a, which is consistent with the local hexagonal packing observed in electron diffraction patterns and high-resolution electron microscopy (HREM) imaging [34]. In Figure 10a, these distinct domain orientations are identified by different colors. The well-aligned parallel chains within the domains form hexagonal structures, while the amorphous interphase is mainly composed of voids and a few disordered DNT chains. Additionally, we plotted the aggregate density as a function of temperature during the cooling process to identify the glass transition temperature (Figure 10b) [35,36]. A clear slope change is observed, with the intersection point indicating a glass transition temperature of 1485 K. This *T*_g_ value is more than 1000 K higher than that of conventional flexible-chain polymers [37,38,39], highlighting the superior thermal stability of DNT aggregates.

Tensile simulation is then performed to study the deformation behavior of the DNT aggregate. A uniaxial tensile load is applied along the y-direction at a strain rate of 10^8^/s in the NPT ensemble, maintaining the temperature at 300 K and the lateral pressures at 1 atm. Figure 10c shows the stress–strain curve upon tensile loading. The Young’s modulus is calculated to be 1.47 GPa by fitting the linear elastic regime of the smoothed curve within a small-strain limit of 0–0.03, which is approximately one order of magnitude higher than that of crystalline polyethylene [40]. Beyond the elastic limit, the aggregate undergoes yielding and buckling, with a yield strength of approximately 64 MPa, which is twice as high as that of crystalline polyethylene [40]. It should be noted that the strain rate accessible in MD simulations is typically several orders of magnitude higher than in experiments due to time-scale limitations. Such high strain rate usually leads to high strain energy and log-dependent increases in tensile modulus and strength of polymer [41,42]. Therefore, the simulated mechanical parameters represent upper-bound estimates, providing a qualitative and relative evaluation rather than a direct quantitative benchmark against quasi-static experimental data. The inset snapshots in Figure 10c reveal the underlying deformation mechanism. The uniaxial tensile deformation is dominated by the cooperative sliding of parallel DNT chains within the aligned crystalline domains. No structural fracture or chain rupture is observed up to a strain of 0.3, demonstrating that the DNT aggregate possesses an exceptional combination of high rigidity and toughness.

The outstanding thermomechanical performance of the DNT aggregate is governed by three primary structural mechanisms: First, the high bending stiffness and short length of individual DNT chains prevent thermal coiling, promoting a dense, parallel hexagonal packing that provides high initial resistance to deformation. Second, the strong intermolecular vdW interactions between parallel DNTs restrict local thermal motion and chain sliding. This strong cohesive energy significantly increases the energy barrier for molecular relaxation, directly yielding the exceptionally high *T*_g_, Young’s modulus, and yield strength. Third, the high mechanical strength of the *sp*^3^-bonded backbone prevents individual chain rupture during tension. Instead, plastic deformation is accommodated by cooperative sliding, while failure propagates exclusively through the low-density amorphous interphases and voids. This failure diffusion mechanism effectively dissipates energy, prevents sudden brittle cleavage, and maintains structural integrity under large strains.

## 5. Conclusions

In summary, robust coarse-grained (CG) potentials for diamond nanothreads (DNTs) have been successfully developed by integrating all-atomistic (AA) simulations with molecular mechanics. The bonded stretching and bending potentials were mapped from continuum beam and rod theories using energy equivalence, while the non-bonded potentials were parameterized via cohesive energy calibrations of parallel DNT pairs. Systematic benchmarking of the interfacial characteristics against AA models demonstrates that a degree of coarse-graining of *r*_0_ = 6Å achieves high physical fidelity. Under this optimal scale, both the radial separation properties (including separation force and vdW energy) and the axial sliding force for the CG models were well reproduced in comparison with those of the AA counterparts under small deformations.

By applying the CG potentials to mesoscale systems, we successfully explored the thermodynamics and large-deformation mechanical behaviors of macroscopic DNT aggregates. Thermodynamic analysis of the discrete annealing process reveals an exceptionally high glass transition temperature (*T*_g_ = 1485 K), which far exceeds those of conventional flexible-chain polymers and highlights the superior thermal stability of DNT networks. Under uniaxial tensile loading, the mesoscale aggregates exhibit a tensile strength comparable to conventional crystalline polymers. Crucially, the large-deformation failure of the aggregates is fundamentally governed by an intermolecular sliding mechanism within parallel crystalline regions, where the damage originates from the amorphous boundaries between crystalline domains rather than individual chain rupture.

## Figures and Tables

**Table 1:** Summary of stiffness and adhesion parameters.

DNT Type	*ρ* (amu/Å)	*EA* (kcal/mol/Å)	*EI* (kcal/mol)	*h*_0_ (Å)	*U_L_* (kcal/mol/Å)
DNT-I	30.94	1142.6	1289.6	6.25	0.8331
DNT-II	36.14	3118.4	4241.0	6.55	0.9874

**Table 2:** Summary of the CG potential parameters for the DNT-I at different *r*_0._

*r*_0_	2	3	4	5	6	7	8
*M* (amu)	61.88	92.82	123.76	154.70	185.64	216.58	247.52
*k_b_* (kcal/mol/Å^2^)	571.30	380.87	285.65	228.52	190.43	163.23	142.83
*k_θ_* (kcal/mol/rad^2^)	644.80	429.87	322.40	257.92	214.93	184.23	161.20
*σ* (Å)	5.64	5.73	5.85	6.00	6.18	6.38	6.61
*ε* (kcal/mol)	0.7582	1.1373	1.5164	1.8955	2.2746	2.6537	3.0328

**Table 3:** Summary of the CG potential parameters for the DNT-II at different *r*_0._

*r*_0_	2	3	4	5	6	7	8
*M* (amu)	72.28	108.42	144.56	180.70	216.84	252.98	289.12
*k_b_* (kcal/mol/Å^2^)	1559.20	1039.47	779.60	623.68	519.73	445.49	389.80
*k_θ_* (kcal/mol/rad^2^)	2120.50	1413.67	1060.25	848.20	706.83	605.86	530.13
*σ* (Å)	5.90	5.99	6.10	6.25	6.42	6.62	6.84
*ε* (kcal/mol)	0.8986	1.3479	1.7972	2.2465	2.6959	3.1452	3.5945

**Table 4:** Summary of CG potential parameters derived for DNT-I and DNT-II.

Functions	Parameters	Units	DNT-I	DNT-II
$U_{\mathrm{CGb}}=\frac{1}{2}k_{b}{\left(r-r_{0}\right)}^{2}$	Equilibrium distance, *r*_0_	Å	6	6
	Tensile stiffness, *k_b_*	kcal/mol/Å^2^	190.43	519.73
$U_{\mathrm{CG}\theta }=\frac{1}{2}k_{\theta }{\left(\theta -\theta_{0}\right)}^{2}$	Equilibrium angle, *θ*_0_	degree	180	180
	Bending stiffness, *k_θ_*	kcal/mol/rad^2^	214.93	706.83
$U_{LJ}\left(d\right)=4\varepsilon \left[{\left(\frac{\sigma }{d}\right)}^{12}-{\left(\frac{\sigma }{d}\right)}^{6}\right]$	Dispersive parameter, *σ*	Å	6.18	6.42
	Dispersive parameter, *ε*	kcal/mol	2.2746	2.6959

## Data Availability

The original contributions presented in this study are included in the article. Further inquiries can be directed to the corresponding authors.
